# Eye-Tracking as a Lens into Expertise Development in Visual Search

**DOI:** 10.1145/3725837

**Published:** 2025-05-22

**Authors:** MEGAN H. PAPESH, MICHAEL C. HOUT, GIOVANNA C. DEL SORDO, BRYAN L. WHITE, ASHLEY P. MATHIS

**Affiliations:** University of Massachusetts Lowell, United States; New Mexico State University, United States; New Mexico State University, United States; New Mexico State University, United States; New Mexico State University, United States

**Keywords:** ACM proceedings, Visual Search, Expertise, Image Perception, Clutter

## Abstract

Visual search professionals frequently examine complex images in which they must locate and identify anomalies (e.g., tumors or lesions in medical images, hostile territories in maps) indicating the presence of important information. This is a difficult perceptual and cognitive challenge that requires years of experience even beyond domain-specific training. We developed a laboratory analogue of this task to examine how people development expertise over time and the changes that occur in visual scanning behaviors as this expertise accrues. Participants’ eyes were tracked as they searched for subtle anomalies during and after different forms of training. Anomaly detection improved with experience and this improvement occurred more quickly in perceptual training conditions. Eye movement analyses revealed that participants’ expertise conferred benefits in both scanning and recognition times. These results suggest that simple perceptual training methods can affect both cognitive and oculomotor components of visual search.

## 1 Introduction

Many skilled professions (e.g., air traffic control, medicine, driving) rely on observers’ ability to quickly extract and identify visual information. Domain-specific expertise in these visual tasks confers perceptual and cognitive benefits that permit observers to better anticipate and respond to visual events [27]. For example, expert referees can efficiently attend to regions of space where penalties are likely to occur while simultaneously (and seemingly continuously) deciding whether one of many possible infractions has occurred [26]. Although experts often receive formal training in their discipline, expert skills are notoriously difficult to verbalize (e.g., [21]), so the behavioral and cognitive changes that emerge with expertise typically result from accumulated experiences rather than direct instruction.

Across many domains, experts typically make faster and more accurate decisions than novices (e.g., [1, 7, 10, 18, 19, 22, 30]). In addition to their improved decision speed and accuracy, experts have also been found to make fewer and more systematic eye movements during their expert task than novices. These differences are observed in many domains, including chess [8, 23, 24], refereeing [25, 26]), and medical image perception (e.g., [12, 17, 20, 30] see [6] for a review), among others. For example, experts spend more time examining regions of space in which there is a high probability of detecting a sought-after item, relative to regions that are more likely to contain distracting or irrelevant information (e.g., [6, 14]). Although this may reflect long-term knowledge of the statistical regularities of their profession, even low-level attentional guidance and visual span are enhanced in experts. In visual search professions (e.g., medical image perception), expert observers are more likely to detect targets pre-attentively [13], locate targets within their first fixation [5], and execute eye movements that span greater distances [5, 21].

Examining expertise in the laboratory typically requires the use of between-groups designs in which experts are compared to trainees or novices within a specific field. For example, researchers studying expertise in medical image perception typically compare working radiologists to those in training (e.g., medical school students, residents) and/or naïve participants (e.g., undergraduate samples; [3, 4, 9, 11]). Despite care taken to equate or control individual differences in factors like visual acuity or age, there remain many possible extraneous variables that may influence results in between-group designs. For example, individuals who possess innate visual skills may self-select into careers that allow them to capitalize on those skills. For that reason, researchers have begun to explore the development of expertise across semi-longitudinal time frames, allowing each participant to serve as both the novice and, later, the expert.

Papesh et al. [21] used a within-groups design to explore the development of visual search expertise across a semi-longitudinal time frame. Novice participants (undergraduate volunteers) committed categories of potential targets to memory and then searched for any representatives from those categories within displays of 32 items while their eye movements were tracked. Displays contained between 0 and 3 target items, and no objects overlapped or occluded others. Across 14 sessions, Papesh et al. found that participants’ target detection accuracy improved and the time they spent viewing individual objects within the display decreased. Coupled with other findings, this led them to conclude that even within-group designs reveal that expertise manifests in improved behavioral performance and more efficient oculomotor behaviors (e.g., scanning metrics, visual span).

**Figure 1:**
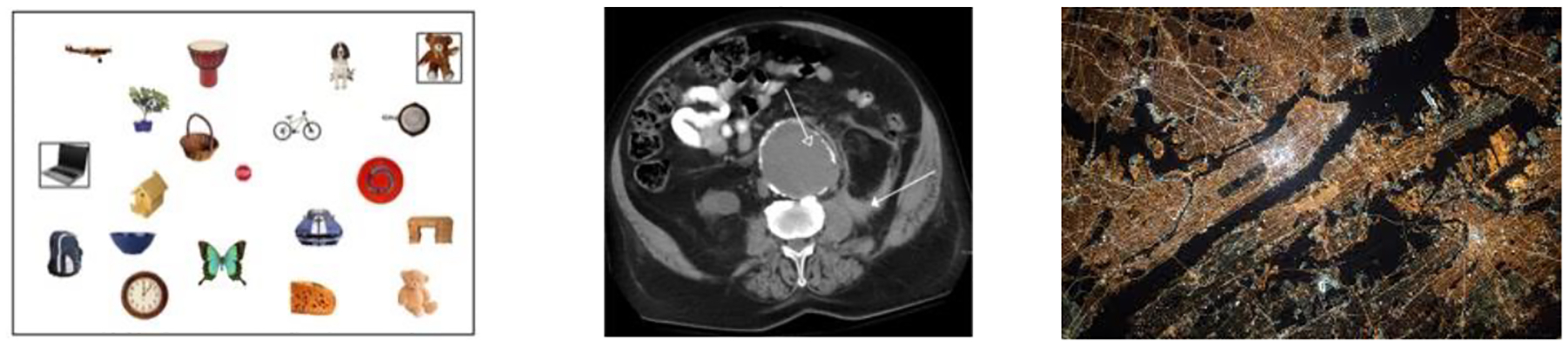
Examples of a typical laboratory visual search array (left; from [21]), an abdominal CT scan (middle; from James Heilman, MD, Wikimedia Commons), and an aerial map (https://pxhere.com/en/photo/919491).

Although Papesh et al. [21] confirmed the results of between-group expertise studies in a within-group design, standard laboratory visual search tasks may not capture the perceptual and cognitive challenges that characterize many expert visual domains. For example, as shown in Figure 1, typical laboratory visual search displays (left) lack the clutter and/or spatial regularity of medical images (center) or maps (right). Unlike search through well-separated objects, medical images and maps are flattened representations of three-dimensional structures. Even volumetric CT scanning still requires observers to scroll through “slices” of tissue, rather than viewing three-dimensional structures in 3D. Additionally, targets in visual search tasks are often well-defined: Observers know the specific objects or categories of objects they seek. In medical image perception or map scanning, observers often have a specific target in mind (e.g., for a patient seen for routine follow-up imaging; a known base or structure in that geographic location), but they must remain vigilant for evidence of secondary targets. Lastly, in visual search studies, targets exist as additions to the array; they are typically unique objects that have no effect on the surrounding items. In medical image perception and map scanning, targets are often malformations of, or additions to, the existing anatomy or landscape, such that their existence can be suggested by their effects on the surrounding parts of the image.

**Figure 2:**
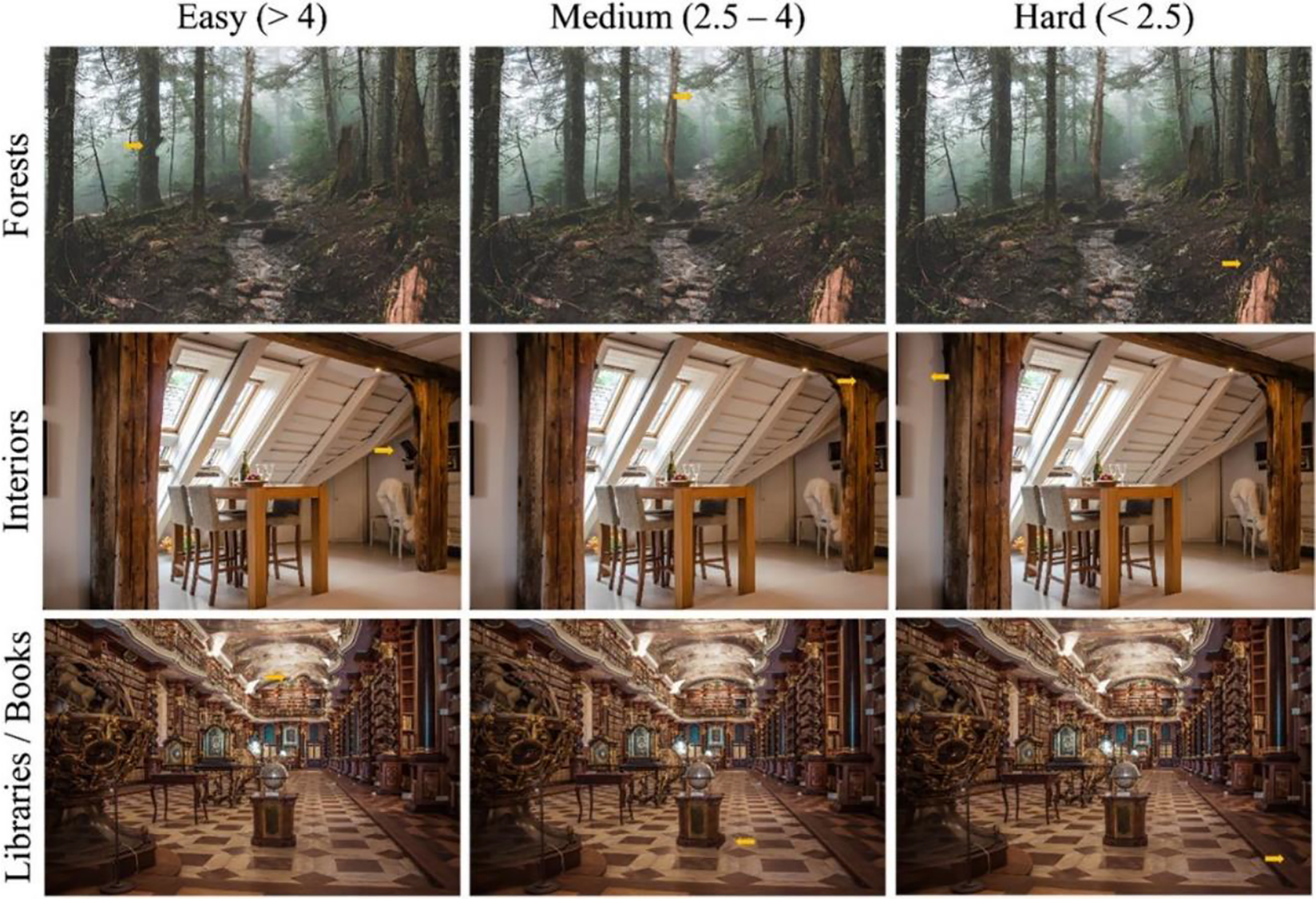
Example scenes from the ODDS Database [15]. Rows indicate different scenes and columns indicate different subtlety ratings for anomalies embedded in those scenes. Embedded yellow arrows indicate the location of anomalous elements.

Given the many differences between typical laboratory search stimuli and applied image screening professions, Hout et al. [15] created a database of images that retain many of the characteristics of medical images but that can nonetheless be searched by novices. The Oddity Detection in Diverse Scenes (ODDS) Database contains images with poorly-specified “oddity” targets embedded in unpredictable locations (along with clean images; see Figure 2). Oddities are ripple deformations which Hout et al. validated using perceptual difficulty ratings and performance metrics.

These validation studies confirmed that the database contains a range of difficulty levels, from barely-perceptible targets to obvious ones, and that naïve observers’ search latencies and detection rates are well-predicted by subtlety ratings. Although the scenes in the ODDS Database lack the structural regularity of medical images or maps and the potential for multiple anomalies to exist within a single image, they address many of the differences between standard visual search stimuli and images in applied contexts. For example, the targets are not nameable or memorizable via category labels, the scenes are perceptually cluttered with many overlapping items, and the targets are malformations of existing visual elements.

### 1.1 The Present Investigation

In the present investigation, we build on research by Papesh et al. [21] to explore how training and expertise affect oculomotor behaviors and performance in a laboratory analogue of searching through images in applied settings (e.g., medicine and map scanning). Specifically, whereas Papesh et al. examined how expertise develops in a visual search task using nameable targets and well-separated, uncluttered displays, we examined whether their effects would replicate in a more perceptually challenging and less-structured search task. We also sought to determine whether error types change with accumulating expertise. For example, observers may fail to identify a target because they simply do not look at it (i.e., a scanning error) or they may look at it, but fail to recognize it as a target (i.e., a recognition error; [16, 28]). To that end, we used stimuli from the ODDS Database [15] to investigate whether perceptual training speeds the development of expertise in oculomotor behaviors and/or performance across a 7-week timespan. We hypothesized that participants would show fewer misses and more systematic/strategic eye movements over time, particularly in objectively difficult trials.

## 2 Method

### 2.1 Participants

**Figure 3:**
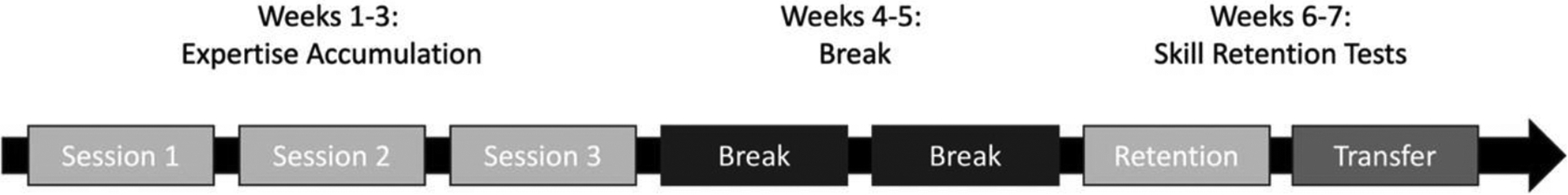
Timeline of the present investigation.

Participants (n = 31) were recruited from the student population of a public university in the southwestern United States and were compensated $15/hr. Participants were required to have normal or corrected-to-normal vision. Participants signed up for once-weekly hour-long sessions during a 7-week period (see Figure 3). For the present study, we restrict our analyses to the expertise accumulation phase to conceptually replicate Papesh et al. [21]. Although no power analysis was conducted to justify the sample size, we report post-hoc power analyses with the Results.

### 2.2 Materials

Stimuli were 284 scenes from the ODDS Database [15], cutouts of target and non-target elements from each scene, and the letters A-Z and numbers 1–9 with and without distortions applied (e.g., a mirror-reversed Z). Stimuli were presented on 24” LCD monitors and eye movements were recorded at 500 Hz using a desktop-mounted SR Systems EyeLink 1000Plus eye-tracker. Experimental procedures were programmed in E-Prime 3.0 software.

### 2.3 Procedure

**Figure 4:**
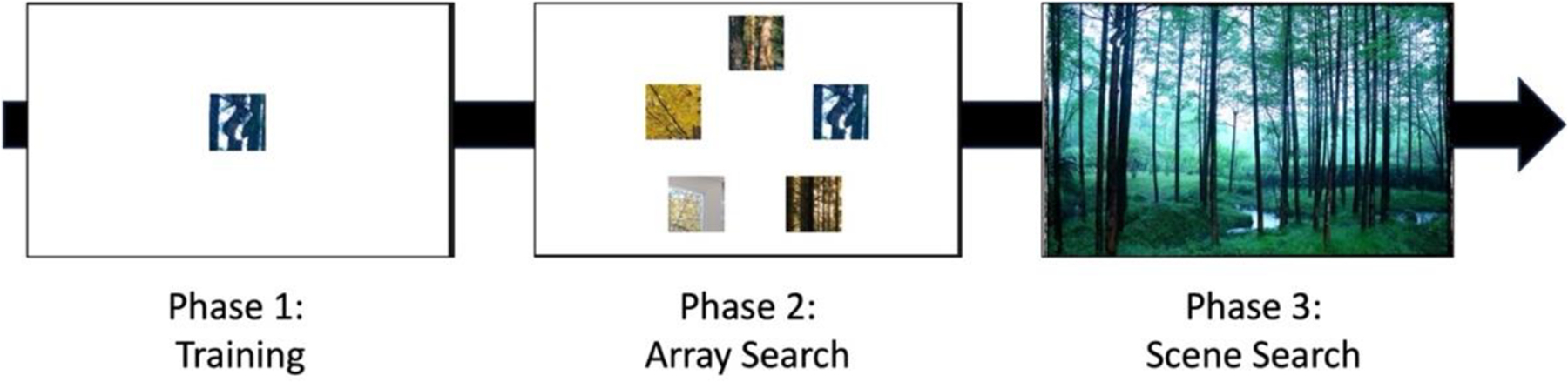
Schematic of each session as participants developed task expertise.

After providing written informed consent, read instructions about the upcoming task and the weekly sessions. Each session began with eye-tracking calibration, using the built-in software with the EyeLink system. All participants were successfully calibrated within two attempts. Each session then followed the same basic structure: A training block, an array search block, and then a scene search block (see Figure 4).

During the training phase, participants completed 192 trials in which they rapidly classified stimuli as containing an anomaly or not. Stimuli were cutouts from ODDS images as anomalous or clean. Half of the trials contained anomalies and half did not. Participants were given accuracy feedback along with a second view of the stimulus to aide learning.

After training, participants completed an array search block consisting of 96 trials. In each trial, which they viewed a circular array containing 5 ODDS image cutouts, each from a different scene in the database. Participants’ task was to rapidly determine whether an anomaly was present or absent. Anomalies appeared in a random subset of 50% of the trials, and the location in the array was also randomized. After each trial, participants received correct/incorrect feedback and were shown the location of the anomalies regardless of their accuracy.

During the final scene search block, participants viewed 96 full ODDS database images one at a time. For each scene, they quickly determined whether a target was present or absent and indicated their response via keyboard. As in previous blocks, they received correct/incorrect feedback.

## 3 Results

### 3.1 Analytic Approach

Because our interest was in the development of expertise, we restrict our focus to sessions 1 through 3, during the development of expertise. A subsequent paper will explore effects related to retention and transfer of skills. Post hoc power analyses for dwell time were conducted for the Array and Scene search tasks. The results indicated strong sensitivity for the Subtlety Rating variable (β = 0.99) and high power for Session (β = 0.890 in the Array task, β = 0.908 in the Scene task), supporting reliable detection of these effects.

Logistic regressions were used for behavioral (miss rate) data and linear mixed-effects models were fit to oculomotor (dwell time) data with participants and session as random effects. Independent variables of session number and scene subtlety rating were added to the model. The best fitting models for each dependent variable are described below.

### 3.2 Behavioral Performance

The best-fitting model for both the array and scene search tasks did not include any interactions (see Tables 1 and 2).

**Figure 5:**
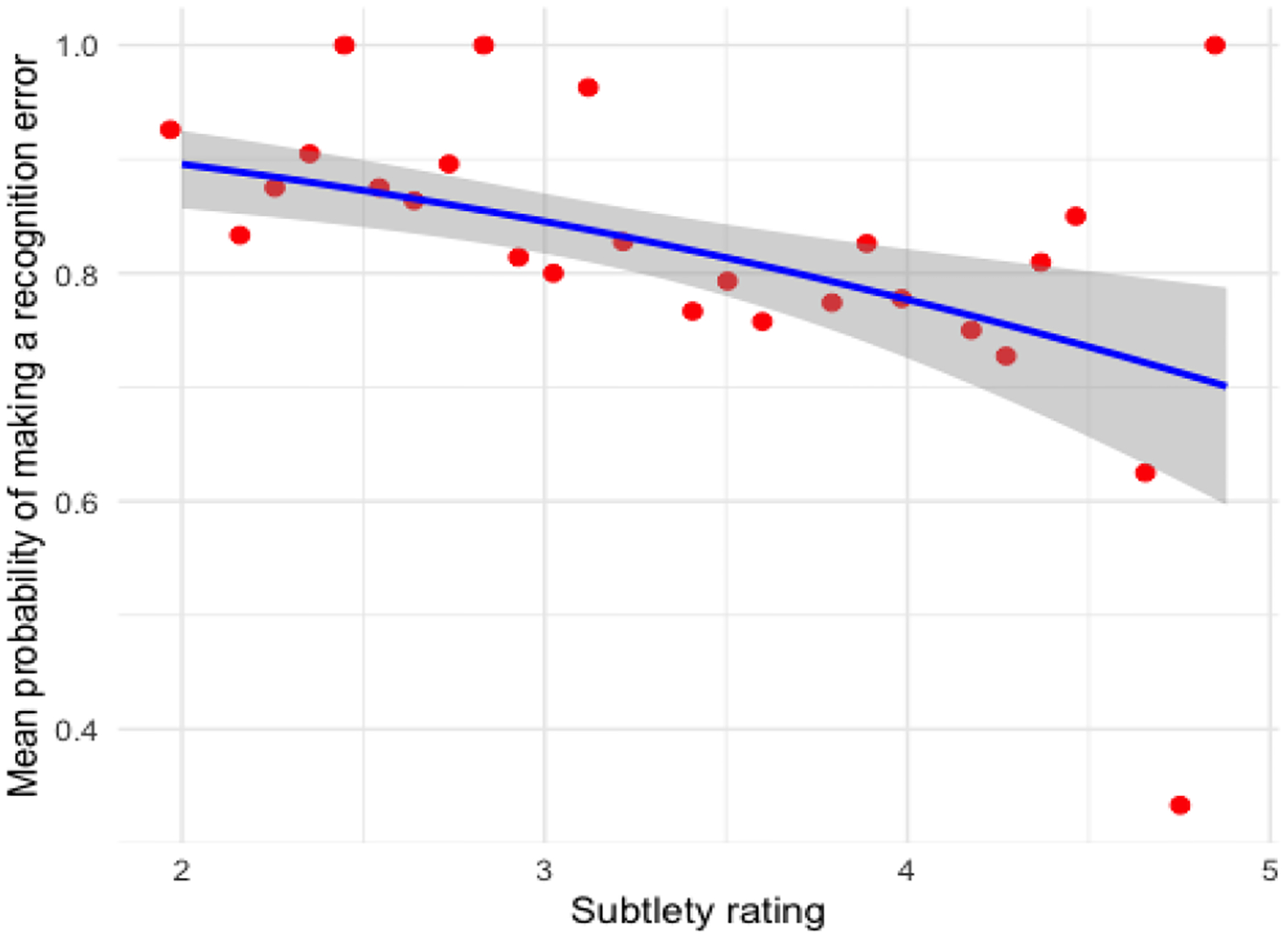
Effect of subtlety rating on the probability of recognition errors in the array search task (blue shading reflects standard error)

**Figure 6:**
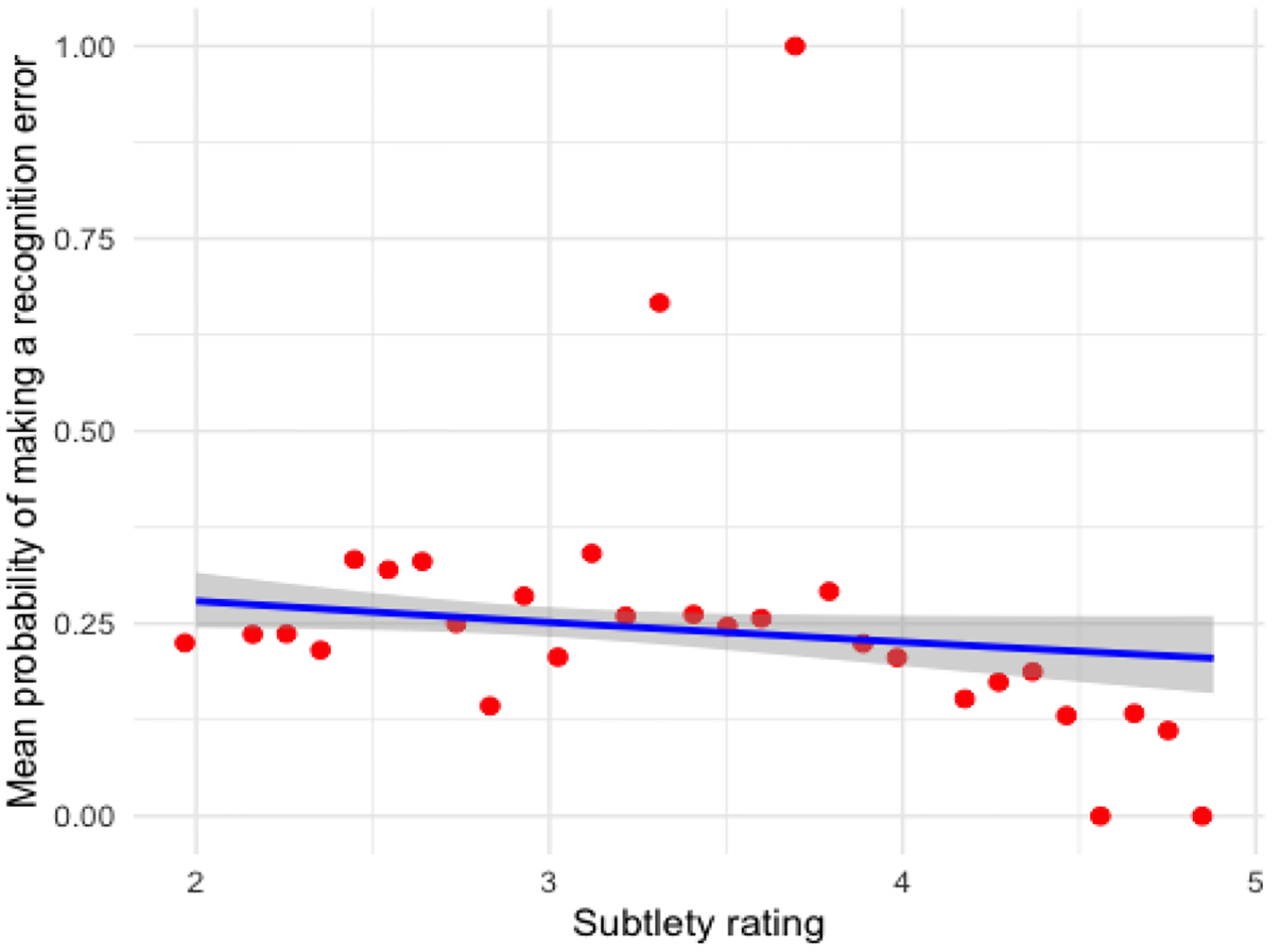
Effect of subtlety rating on the probability of recognition errors in the scene search task (blue shading reflects standard error)

In the array search task, recognition errors (83.8%) were more frequent than scanning errors, and the subtlety of the anomaly in the scene was associated with miss rates, such that more subtle anomalies led to increase in scanning errors (see Figure 5). In the scene search task, scanning errors (74.9%) were more common than recognition errors, with subtle anomalies showing a marginal association with increased scanning errors (see Figure 6). Contrary to our predictions, no effects were observed related to Session in either task, indicating that participants’ behavioral performance did not change much across sessions 1 through 3.

### 3.3 Oculomotor Measures

The best-fitting model did not include interactions (see Tables 3 and 4).

**Figure 7:**
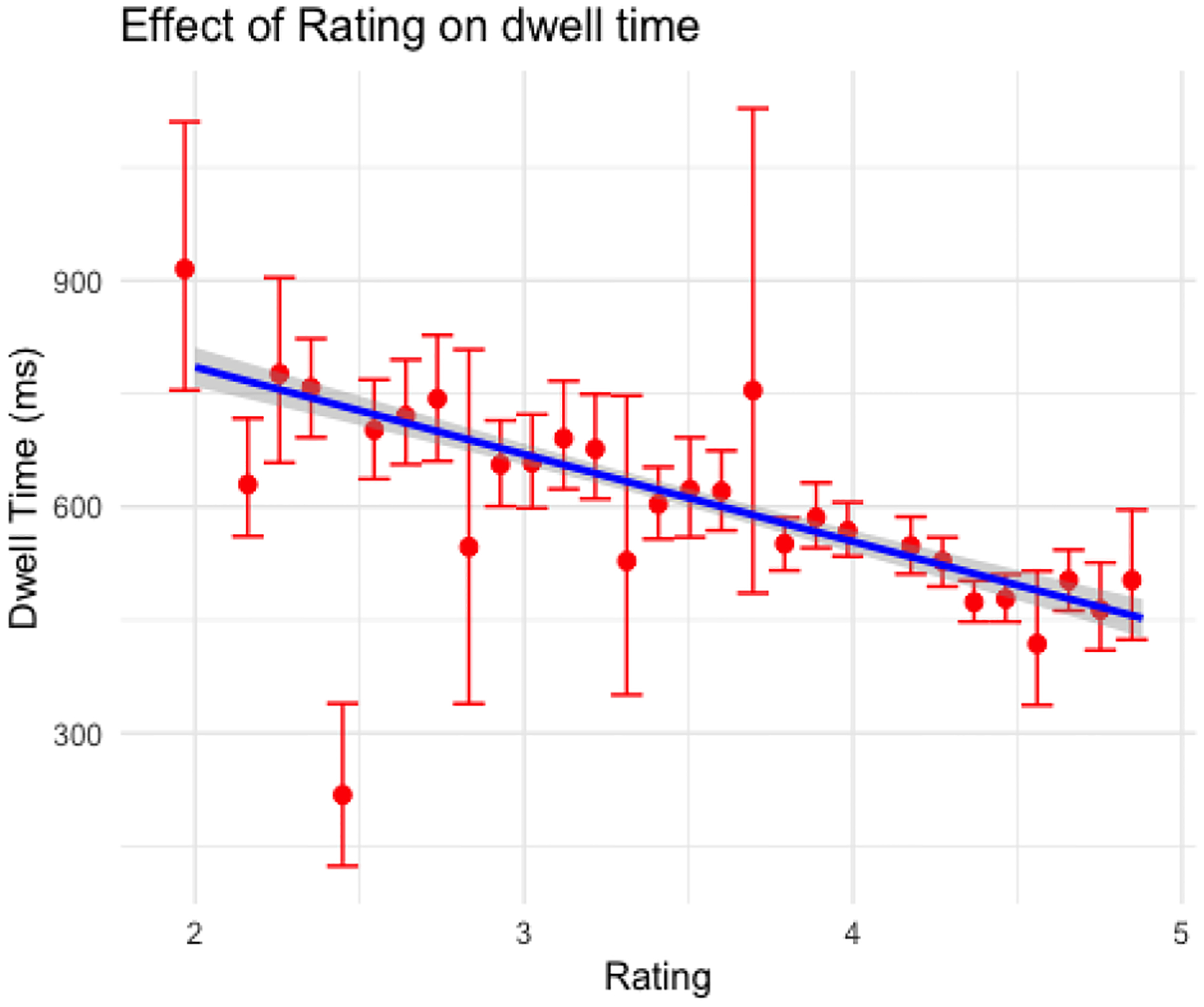
Effect of subtlety rating on dwell time in the array search task (error bars depict standard error)

**Figure 8:**
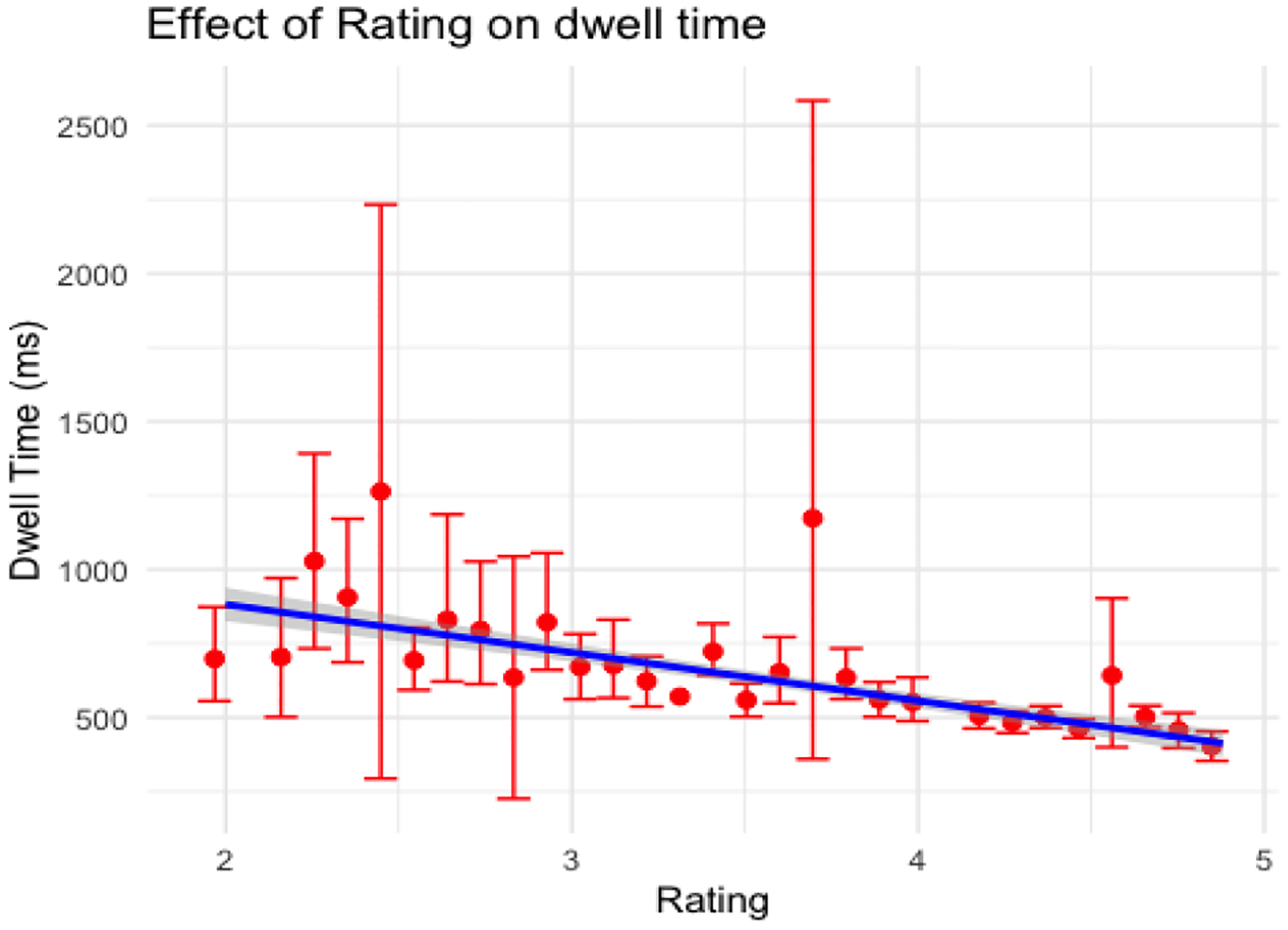
Effect of subtlety rating on dwell time in the scene search task (error bars depict standard error)

In both versions of the search task, more subtle anomalies were associated with increased dwell times (see Figures 7 and 8), but expertise manifest as decreasing dwell times across sessions.

## 4 Discussion

In this experiment, participants experienced domain-specific perceptual learning across three weeks of experience with an anomaly search task. Participants’ behavioral performance revealed that recognition errors (sometimes called “look but fail to see errors,” [28]) were more common in the array-search task, but scanning errors (i.e., errors in which participants did not look at the target) were more common in the more realistic scene search task. Although behavioral performance did not reveal much change as a function of accumulating experience, the eye movement analyses revealed that participants’ performance was becoming more expert-like by their third session. With experience, participants’ dwell times (i.e., the time they spent inspecting areas of the visual display while their eyes were relatively stable) gradually decreased, reflecting an increased ability to recognize both anomalous and non-anomalous areas of the display.

Although these results are suggestive about the role of experience and perceptual learning in anomaly search, they do not fully capture expertise development. For example, participants in the present study completed perceptual learning and training on the same images used to evaluate their expertise. In an ideal design, expertise would be measured within and across tasks, allowing for greater inferences about the cognitive abilities shaped by the training. Additionally, the stimuli used in the present research lack the structural regularity and context-based clues that may be present in certain forms of complex visual search (e.g., medical image perception). Because few studies in the visual search literature have utilized unnamable “anomaly” targets in visually complex ways, the present results represent a preliminary step toward better understanding how eye movement patterns change with experience.

These results have implications for future studies into expert visual search (e.g., map scanning) or simulated medical image perception. In addition to providing proof-of-concept data showing that perceptual training can speed the development of expertise in certain forms of complicated visual search, the data reveal that expertise effects can be observed in the oculomotor record before they emerge in the behavioral data. These results are consistent with prior studies examining expertise-induced changes in eye movements (e.g., [21]) and studies showing that perceptual experience can enhance both target and distractor recognition (e.g., [31]). Future studies can use eye movements to quantify the development of expertise and potentially as a strategy for instructional intervention.

By monitoring participants’ eye movements as they accumulated experience and expertise, were found that changes in their scanning behaviors emerged before changes in their performance metrics (e.g., accuracy). To our knowledge this is the first study to demonstrate the timecourse of when these expert-like behaviors emerge in a semi-longitudinal study.

## Figures and Tables

**Table 1:** Logistic regression results for a model predicting recognition to scanning miss rates in the array search task in sessions 1 through 3

Predictor	Estimate	SE	z-value	P
Intercept	2.99	0.45	6.72	<.001
Subtlety Rating	−0.45	0.13	−3.51	<.001
Session 2	0.13	0.23	0.55	.582
Session 3	0.03	0.25	0.12	.903

a Significant predictors are shown in bold.

**Table 2:** Logistic regression results for a model predicting recognition to scanning miss rates in the scene search task in sessions 1 through 3

Predictor	Estimate	SE	z-value	P
Intercept	−0.54	0.25	−2.16	.031
Subtlety Rating	−0.15	0.08	−1.89	.059
Session 2	−0.15	0.13	−1.18	.237
Session 3	−0.21	0.13	−1.69	.092

a Significant predictors are shown in bold.

**Table 3:** LMM results for a model predicting dwell times in the array search task in sessions 1 through 3

Predictor	Estimate	SE	t-value	P
Intercept	1072.47	54.66	19.62	<.001
Subtlety Rating	−118.39	7.28	−16.27	<.001
Session 2	−66.42	31.34	−2.12	.043
Session 3	−79.82	35.41	−2.25	.032

a Significant predictors are shown in bold.

**Table 4:** LMM results for a model predicting dwell times in the scene search task in sessions 1 through 3

Predictor	Estimate	SE	t-value	P
Intercept	1207.75	66.75	18.09	<.001
Subtlety Rating	−151.75	15.82	−9.59	<.001
Session 2	−71.98	28.15	−2.56	.010
Session 3	−84.64	28.47	−2.97	.002

a Significant predictors are shown in bold.

## CCS Concepts:

• **Applied computing** → **Psychology**
